# Impact of Electronic Health Records on Information Practices in Mental Health Contexts: Scoping Review

**DOI:** 10.2196/30405

**Published:** 2022-05-04

**Authors:** Timothy Charles Kariotis, Megan Prictor, Shanton Chang, Kathleen Gray

**Affiliations:** 1 School of Computing and Information Systems University of Melbourne Parkville Australia; 2 Melbourne School of Government The University of Melbourne Carlton Australia; 3 Melbourne Law School University of Melbourne Carlton Australia; 4 Centre for Digital Transformation of Health University of Melbourne Parkville Australia

**Keywords:** electronic health records, psychiatry, mental health, electronic medical records, health informatics, mental illness, scoping review, clinical decision support

## Abstract

**Background:**

The adoption of electronic health records (EHRs) and electronic medical records (EMRs) has been slow in the mental health context, partly because of concerns regarding the collection of sensitive information, the standardization of mental health data, and the risk of negatively affecting therapeutic relationships. However, EHRs and EMRs are increasingly viewed as critical to improving information practices such as the documentation, use, and sharing of information and, more broadly, the quality of care provided.

**Objective:**

This paper aims to undertake a scoping review to explore the impact of EHRs on information practices in mental health contexts and also explore how sensitive information, data standardization, and therapeutic relationships are managed when using EHRs in mental health contexts.

**Methods:**

We considered a scoping review to be the most appropriate method for this review because of the relatively recent uptake of EHRs in mental health contexts. A comprehensive search of electronic databases was conducted with no date restrictions for articles that described the use of EHRs, EMRs, or associated systems in the mental health context. One of the authors reviewed all full texts, with 2 other authors each screening half of the full-text articles. The fourth author mediated the disagreements. Data regarding study characteristics were charted. A narrative and thematic synthesis approach was taken to analyze the included studies’ results and address the research questions.

**Results:**

The final review included 40 articles. The included studies were highly heterogeneous with a variety of study designs, objectives, and settings. Several themes and subthemes were identified that explored the impact of EHRs on information practices in the mental health context. EHRs improved the amount of information documented compared with paper. However, mental health–related information was regularly missing from EHRs, especially sensitive information. EHRs introduced more standardized and formalized documentation practices that raised issues because of the focus on narrative information in the mental health context. EHRs were found to disrupt information workflows in the mental health context, especially when they did not include appropriate templates or care plans. Usability issues also contributed to workflow concerns. Managing the documentation of sensitive information in EHRs was problematic; clinicians sometimes watered down sensitive information or chose to keep it in separate records. Concerningly, the included studies rarely involved service user perspectives. Furthermore, many studies provided limited information on the functionality or technical specifications of the EHR being used.

**Conclusions:**

We identified several areas in which work is needed to ensure that EHRs benefit clinicians and service users in the mental health context. As EHRs are increasingly considered critical for modern health systems, health care decision-makers should consider how EHRs can better reflect the complexity and sensitivity of information practices and workflows in the mental health context.

## Introduction

### Background

Electronic health records (EHRs) are being adopted in many health systems to improve the collection, sharing, and use of health care information [1]. Such information practices play a critical role in providing safe and high-quality care [2,3]. EHRs promise more integrated and connected health services, which are recognized by the World Health Organization and many governments as essential for sustainable, effective health systems [4-6]. Owing to the complex array of services that support service users, the fragmentation of care and limited information sharing are common in the mental health context [7]. Limited information sharing among health care services affects the planning and provisioning of appropriate care, such as medication management and reconciliation [8,9]. It can also negatively affect service users’ experience of mental health care, especially when it leads to them having to retell their stories multiple times [10]. However, information sharing also comes with risks for service users, such as the stigma associated with mental health conditions [11]. Thus, mental health information tends to be considered highly sensitive information, requiring extra protection [12].

Information is critical to modern health care, especially mental health care, and health records are vital tools for documenting, organizing, and using information [8,13]. When health care professionals provide care to service users, they undertake a range of information practices, including seeking, using, documenting, and sharing information [14]. Health records play a critical role in such practices. Coiera [15] outlined that a health record has many functions, including enabling communication among staff through the information in the record, providing a central source of information for care, acting as an informal workspace for capturing ideas, and being a historical archive that can inform future care. Mental health records are especially complex because many entries can be included in the record [16,17].

EHRs are a core health informatics tool for the improvement of health care quality, partly through improved information quality and accessibility [15]. EHRs are, in one sense, a digitized version of the health care record but are also much more in that they introduce new practices and workflows [18-21]. For example, EHRs have been found to affect how information is documented in clinical records by introducing structured data entry forms and disrupting the collection of narrative information [22-25]. Internationally, the uptake of EHRs in the mental health context has been much slower than in other health contexts [26-29]. A recent scoping review on the effective implementation of electronic medical records (EMRs) in mental health settings also identified limited research on this topic [30]. Apart from the barriers faced by all health settings in adopting EHRs, such as interoperability, time impacts, and workflow changes, there may be particular issues in the mental health context that require investigation [31].

Information sharing relies on a range of information behaviors and practices by clinicians and service users [32,33]. Information behavior has been used to capture the range of human behaviors related to seeking and using information [34]. In comparison, information practice considers how information behaviors are embedded and shaped by organizational contexts and interactions [34]. Østensen et al [35] defined information practice as “a socially constructed practice that determines how information is produced, organised, disseminated, distributed, reproduced and circulated in the community, and which specific types of information are legitimized.”

Going forward, we purposively use the term *information practice* rather than the more widely used term *information behavior*. Adopting this language aligns with our understanding that social and organizational rules and norms shape how clinicians practice information sharing [36-40]. Using the concept of information practice allows us to reflect on how particular issues in the mental health context, such as sensitive information and stigma, influence information practices.

Mental health care involves various sensitive information practices, such as people sharing a range of sensitive and potentially stigmatizing information, from personal trauma to behavioral patterns [9,41]. This information can also be considered stigmatizing, both publicly and within health care settings. Stigma is a common theme across a number of studies exploring the experiences of service users in the mental health context [42-44]. For example, it has been found that people with diagnoses such as borderline personality disorder experience stigma from health professionals, which affects their care [45]. Health care professionals in the mental health context are also aware of the sensitivity of mental health information [9,46]. Several commentaries have raised concerns about how sensitive information is recorded in EHRs and its implications for privacy and security [47-50].

The documentation of mental health information is another information practice that is an issue in EHR use in the mental health context. Mental health services are more likely to rely on narrative information [51]. For example, Kobus et al [51] pointed out that although most medical conditions rely on quantitative measures, depression relies partly on reviewing narrative progress notes. However, one of the reasons for adopting an EHR is to standardize data collection through structured data fields [24]. The lack of standardized information formats in the mental health context is a potential barrier to EHR uptake [52,53]. There is also great diversity in how mental health information is documented and used across professions, which complicates the standardization of mental health information [54]. Although diagnostic codes are available for mental health conditions, it is not easy to establish a clear diagnosis and associated diagnostic codes in the mental health context [55-57].

A final issue that has been raised in the literature as a concern for the adoption of EHRs in mental health contexts is the impact it could have on the therapeutic relationship [58,59]. Therapeutic relationships are critical for providing mental health care [60,61]. Adding an EHR to clinical encounters, which may bring new information practices, has been raised in different care settings as a potential barrier to establishing and maintaining a therapeutic relationship [62]. Shank et al [63] found that mental health clinicians worried that EHRs would divert their attention from service users and negatively affect the therapeutic relationship.

Research on the use of EHRs in the mental health context is at a low stage of maturity, with a diverse array of studies responding to different contextual issues. Thus, a scoping review is necessary to understand the literature [64]. This scoping review aims to identify the impact of EHRs, implemented in the mental health context, on information practices. Furthermore, it aims to explore how, in the use of EHRs, sensitive information, data standardization, and impacts on the therapeutic relationship have been considered, if at all.

The review had the following objectives and research questions:

1. In mental health contexts, what impact do EHRs have on information practices, and how do these changes affect other aspects of care?
2. In mental health contexts, how have sensitive information, data standardization, and therapeutic relationships been managed when using EHRs?

### A Note on Language

We chose to use the term *service user* to represent people accessing and using mental health services and chose not to use terms such as *client* as this suggests that people voluntarily use services, which is not always the case in mental health contexts. Terms such as *patient* can be considered as disempowering for people who access services. We acknowledge that the terminology in this space is not settled and that others may consider different terms more appropriate.

The title of this paper refers to the *mental health context*. We chose this term to capture the broad range of clinical and nonclinical services that people may access when experiencing mental health issues [65].

Throughout this paper, we have raised terms such as *mental health data* and *mental health information*. These terms are not clearly defined in the literature, and we will return to this issue in the *Discussion* section.

## Methods

### Overview

The scoping review is a method of synthesizing research and can support various methods, objectives, and study types [64,66,67]. Unlike systematic reviews, scoping reviews do not attempt an exhaustive review of all relevant studies but rather aim for a breadth of evidence. Owing to the relatively recent uptake of EHRs in mental health care, it is appropriate to conduct a scoping review of this emerging evidence to consider a broad definition of EHRs and a range of study types.

This scoping review was informed by the Arksey and O’Malley [67] framework for scoping reviews. We were also informed by the PRISMA-ScR (Preferred Reporting Item for Systematic Reviews and Meta-Analyses extension for Scoping Reviews) checklist and explanation [68]. However, some criteria were not relevant to our study because of the thematic synthesis approach we used to analyze the included studies. Our approach to this scoping review has been to explore the literature on EHRs and describe what it tells us about the impact of EHRs on information practices in the mental health context. Unlike some scoping reviews, we chose not to map the trends in the literature. As different jurisdictions are moving at different speeds in their adoption of EHRs, and due to the breadth of the topic, we did not view the mapping of trends as feasible or helpful in this specific review.

### Inclusion and Exclusion Criteria

#### Overview

We included studies that have examined EHRs in the adult mental health context, either by being based in mental health settings or being used by or for people with a mental health diagnosis. Nonclinical services (eg, housing services) providing services to people with mental illness were also included in this review, in keeping with the definition of health as “a state of complete physical, mental and social well-being and not merely the absence of disease or infirmity” [69].

We included studies that mentioned using EMRs, EHRs, or any associated terms such as health information systems. Häyrinen et al [70], in a review of the literature, found that there are many terms used to describe EHRs, with various functions, formats, users, settings, and purposes. We acknowledge that EHRs, EMRs, and other terms are different but interlinked systems. An EMR is generally considered to be a record of a person’s health encounters in a specific health setting. In contrast, an EHR is usually a compilation of summary information from across EMRs in a region, country, or health system [71]. However, these definitions are not always made clear or defined in the literature, and thus, we did not adopt a specific definition in this paper. There is no one gold standard definition of an EHR or EMR, with peak health informatics organizations using the same definition for both terms [72]. Going forward, we have used the term EHR as an umbrella term to represent the information systems used to manage service users’ health information by and for health services.

This review included any primary evidence that explored the use of EHRs in the mental health context published before April 2021. We excluded studies that focused on children’s health care in acknowledgment that this field raises several unique issues, such as the involvement of parents, which is worthy of a specific review. We excluded studies during the full-text screening that were not relevant to EHRs, the mental health context, or information practices. Studies that focused on clinicians’ perceptions of EHRs in general rather than the EHR that was implemented were also excluded. The case studies were evaluated on a case-by-case basis, depending on the level of detail provided. We excluded studies that described only the design and development of an EHR.

Several types of EHRs provide service users access to their health information, such as personal EHRs, patient portals, and initiatives such as OpenNotes. We excluded these from this review as they raise unique issues regarding how service users access and use their health information. We acknowledge that systems such as OpenNotes will have implications for our study questions. However, we consider these systems to be more thematically aligned with patient portals and personal EHRs, which would benefit from a separate review. Readers interested in this topic should read the recent scoping review by Zhang et al [73] on the use of patient portals in mental health settings.

#### Types of Studies, Information Sources, and Search Strategy

Embase, Scopus, and PsycINFO were searched using a combination of key terms, an example of which is provided in Textbox 1. The search strategy was developed iteratively alongside the identification of key terms in the literature and hand searching of reference lists. This search was initially undertaken in late 2018 and then updated in December 2020, with new papers continually identified until April 2021, when the final draft was completed. No date limitation was applied in the initial search as we wanted to identify all relevant health informatics literature, which ranged across several decades [74]. Papers not published in English were excluded. The first author (TCK) read a subset of articles from the initial search to develop further search terms, which were then applied across PubMed, CINAHL, SocINDEX, and Web of Science. We also searched research repositories: Google, Google Scholar, Grey Literature Report, TROVE, OPEN Grey, and Social Care Online.

**Example search strategy run on Embase.**

**Search number and search term**

1. Electronic health record/
2. Electronic medical record*
3. Electronic patient record*
4. EHR
5. EPR
6. Health information system
7. Health Information Exchange
8. Mental Disease
9. Mental Illness
10. Mental health care
11. Behavio?l health care
12. Mental health service*
13. 1 OR 2 OR 3 OR 4 OR 5 OR 6
14. 7 OR 8 OR 9 OR 10 OR 11
15. 12 AND 13

#### Study Selection

**Figure 1:**
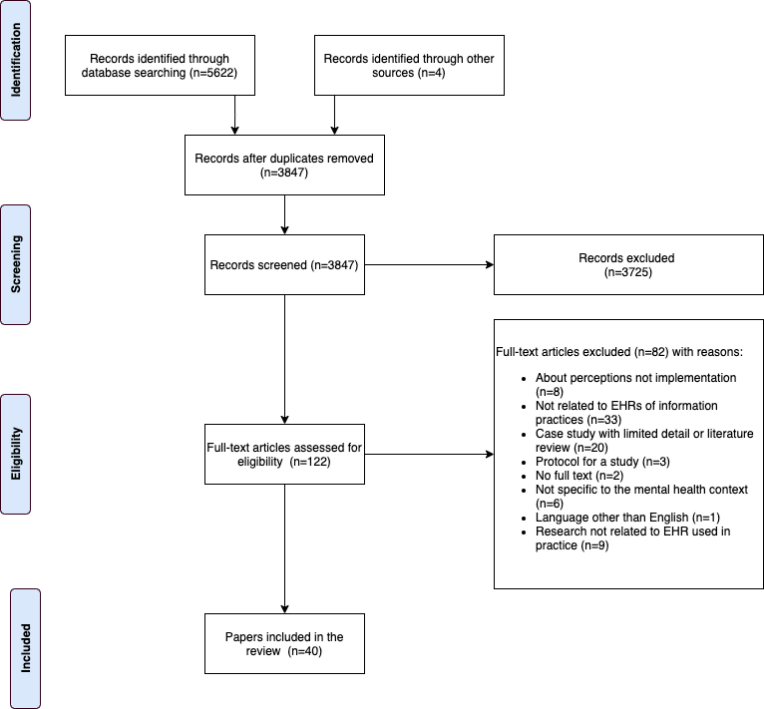
PRISMA (Preferred Reporting Item for Systematic Reviews and Meta-Analyses) diagram. EHR: electronic health record.

We identified 3847 nonduplicate articles. The titles and abstracts were screened against the inclusion criteria by TCK. Approximately 3.17% (122/3847) of articles were considered potentially relevant and were retrieved from the full text. TCK reviewed all 122 full-text articles, with SG and MP each independently reviewing half of the full-text articles. Differences were resolved by discussion and mediation by KG. Of the 122 articles reviewed in the full text, 82 (67.2%) were excluded, and 40 (32.8%) were included (Figure 1).

#### Charting the Results

To provide an overview of the study characteristics, we charted the objectives, study design, research method, study participants, country, study setting, EHR description, year of publication, and theories used in the included studies in a spreadsheet.

Owing to the breadth of the study types and objectives, covering a range of qualitative and quantitative methodologies, we followed Lakshman et al [75] in adopting both a textual narrative and thematic synthesis approach to analyzing the included studies. The textual analysis involved tabulating the study findings alongside their characteristics and conclusions. We adopted a thematic synthesis approach to analyze the qualitative papers included in this review. This method involved coding the text, developing descriptive themes, and generating analytical themes [76,77]. Following the method by Thomas and Harden [76], we initially developed descriptive themes by coding both direct participant quotes and researcher interpretations. We approached this by free-coding findings in an unstructured mind map, which we used to develop descriptive themes. Our research questions framed this coding process so that we coded anything related to information practices or the issues outlined in the *Introduction* section. We also considered the factors that shaped the way information was collected or shared in the presence of an EHR or the outcomes of changes in information practices. We intended for these descriptive themes to stay as close to the original findings as possible.

Next, we used our review questions to develop the analytical themes. We integrated the quantitative data we had extracted during the textual analysis during this process. Thomas and Harden [76] described this process as potentially controversial as it relies on the researcher’s judgment and insight. This iterative process aimed to capture the descriptive data developed in the initial analysis. The analysis identified 6 major themes describing the impact of EHRs on information practices in the mental health context. Of these themes, 5 had subthemes that explored specific topics relevant to the theme***.***

### Study Characteristics

#### Overview

The studies included in this review were highly heterogeneous. This heterogeneity reflects one of the strengths of a scoping review in that it was inclusive of many study types. The following sections describe the characteristics of the included studies. Owing to the heterogeneity of study types and limited use of standardized terms, comparisons between studies were limited. Table 1 outlines the key study characteristics.

**Table 1:** Study characteristics (N=40).

Characteristics			Values, n (%)
**Study design**			
	Quantitative	21 (53)	
	Qualitative	11 (28)	
	Mixed methods	8 (20)	
**Research method^a^**			
	Surveys	15 (38)	
	Interviews or focus groups	7 (18)	
	Chart reviews	5 (13)	
	Cross-sectional or secondary data use	5 (13)	
	Quality improvement	3 (8)	
	Ethnographic or observational	6 (15)	
	Descriptive case studies	1 (3)	
**Study sample**			
	Clinicians or health care professionals	26 (65)	
	Administrator, IT^b^, or management	9 (23)	
	Service users	4 (10)	
	No participants (eg, record review)	13 (33)	
**Countries**			
	United States	27 (68)	
	United Kingdom	8 (20)	
	Canada	2 (5)	
	Other	3 (8)	

^a^Some studies included multiple methods and thus were counted twice.

^b^IT: information technology.

#### Study Design and Research Method

A range of study designs and research methods were represented in the included studies. Most were quantitative (21/40, 53%) [78-98], with qualitative (11/40, 28%) [99-109] and mixed method studies (8/40, 20%) [110-117] also included. We categorized studies based on the broad category of research methods, including surveys (15/40, 38%) [78,80-85,89,92,95-97,110,113,116], qualitative interview/focus group studies (7/40, 18%) [100-104,110,111], chart review of specific EHRs (5/40, 13%) [85-87,114,117], cross-sectional analysis of EHR data or comparison with other secondary data (5/40, 13%) [90,91,93,95,98], quality improvement initiatives (3/40, 8%) [79,88,111], ethnographic or observational (6/40, 15%) [99,105-108,112], and descriptive case studies (1/40, 3%) [109].

The objectives of the included studies varied. We compared the objectives and research questions of the included studies and grouped them according to similar topic areas, as outlined in Table 2 (some studies had multiple objectives). We also included the publication years in Table 2 to showcase how certain topics were not confined to any specific period.

**Table 2:** Topics of included studies and related publication dates.

Topics of included studies	Publication years of included studies
Exploring the adoption of EHRs^a^ in the mental health care context	2015 [116] and 2018 [94]
Evaluation of an EHR implementation	2009 [78], 2010 [107], 2011 [108], 2012 [99], 2017 [79], and 2018 [110]
Exploring the use of EHRs to provide mutual access to psychiatric records	2013 [80] and 2015 [81]
Exploring the impact of EHRs on the therapeutic relationship or person-centered care	2010 [82], 2011 [84] 2017 [101], 2019 [111], 2020 [83], and 2020 [85]
Exploring the use of EHRs in integrated or collaborative care contexts	2012 [113], 2012 [113], 2015 [112], 2015 [81], and 2018 [86]
Comparing documentation in EHRs with documentation in paper records	2007 [87], 2016 [88], and 2018 [114]
Exploring service users’ experiences or satisfaction with care when an EHR is present	2018 [110] and 2020 [90]
Exploring the barriers, facilitators, workarounds, and usability of EHRs in the mental health context	2010 [103], 2011 [108], 2012 [113], 2012 [99], 2013 [109], 2014 [100], 2015 [116], 2015 [112], 2017 [115], 2017 [101], and 2021 [102]
Exploring the impact of EHRs on health care professionals’ information practices and behavior	2004 [105], 2010 [106], and 2016 [104]
Exploring clinicians’ satisfaction and perspectives of EHRs	2009 [89], 2015 [92], and 2018 [110]
Exploring information availability or documentation of specific diagnoses in EHRs	2013 [117], 2016 [91], 2016 [95], 2016 [96], 2019 [93] 2020 [98], and 2020 [97]

^a^EHR: electronic health record.

#### Participants

In most studies that involved direct data collection from human participants, such as EHR evaluations, the participants were health care professionals. The type of health care professional was not always reported or was generalized as *medical professionals*. Overall, primary health care clinicians, physicians, psychiatrists, psychologists, social workers, and nurses were well-represented across the studies. Some studies (9/40, 23%) included administrative, management, or information technology staff [78,97,99-103,108,112]. Only 10% (4/40) of studies involved service users [82,83,108,110]. Table 3 provides more details regarding the types of participants in the included studies.

**Table 3:** Participant roles reported in included studies (N=40).

Participant role	Included studies reporting this role, n (%)	Reference
Primary health care professional	4 (10)	[81,97,110,112]
Physician	6 (15)	[92,99,102,106,107,115]
Psychiatrist	7 (18)	[80,89,96,99-101,103]
Psychologist or psychology technicians	9 (23)	[78,89,92,96,103-105,111,116]
Behavioral health clinicians or mental health clinicians	5 (13)	[83,101,102,110,112]
Nurse, psychiatric nurse, or nurse practitioner	11 (28)	[78,84,89,96,99,100,102,103,105,114,115]
Social workers or social assistants	7 (18)	[92,96,100,103-105,111]
Pharmacists	3 (8)	[78,99,102]
Other allied health professionals	5 (13)	[99,100,105,107,111]
Other clinical or health care staff	12 (30)	[78,85,89,96,97,99-101,107,108,110,111]
Administrative staff	5 (13)	[78,102,103,107,112]
Information technology staff	4 (10)	[97,99,100,108]
Implementation teams	4 (10)	[99,100,107,108]
Service users	4 (10)	[82,83,108,110]
No participants (eg, secondary data and chart review)	13 (33)	[79,86-88,90,91,93-95,98,109,113,117]

#### Countries

Most studies took place in the United States (27/40, 68%) [80-83,85-90,92-98,101,102,104,106,110-113,115,116], followed by the United Kingdom (8/40, 20%) [78,84,99,100,105,107,108,117]. Canada (2/40, 5%) [79,114], France (1/40, 3%) [103], Brazil (1/40, 3%), and Ireland (1/40, 3%) [91] were also represented in the included studies. There were no clear differences in the approaches or methods across jurisdictions. The limited number of studies in countries outside the United States and the heterogeneity of study types limited any comparison.

#### Settings

A variety of health care settings were represented in the included studies, ranging from psychiatric hospitals to community mental health settings. The type of setting was not reported to support easy comparison. These settings are outlined in Multimedia Appendix 1 [54,77-113,115,116] using terminology from the included studies.

#### Year of Publication

**Figure 2:**
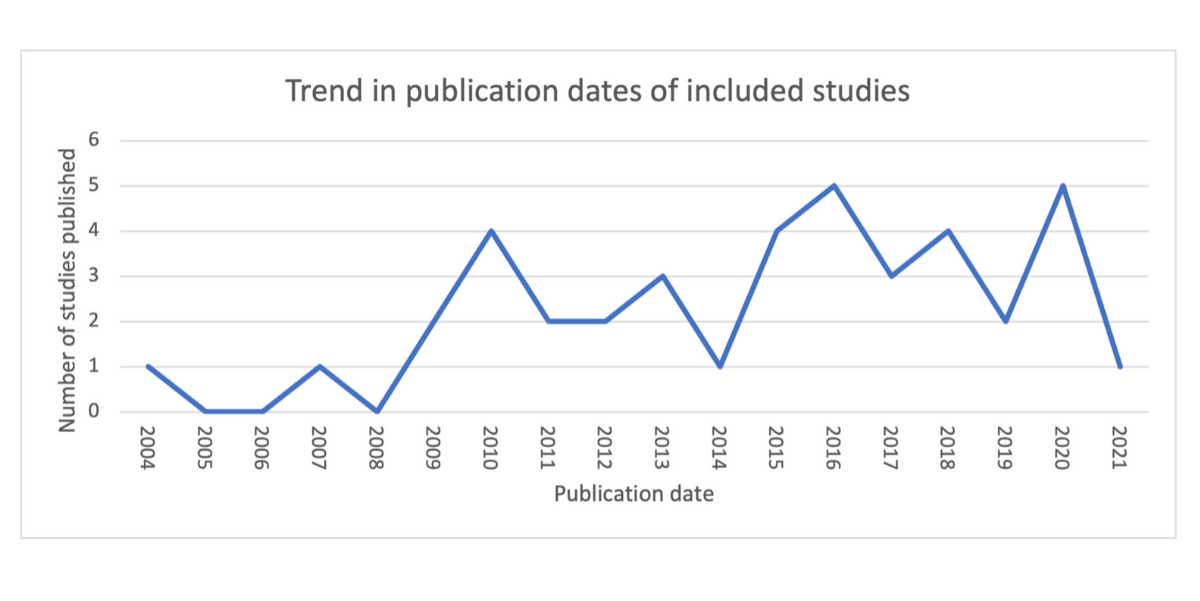
Trend in publication year of the included studies.

The included studies ranged in publication date from 2004 to 2021 (Figure 2). Although our search strategy had no date restrictions, the terminology used in the search may have shaped what studies were included. Older systems such as computerized patient records may not have been identified. This search strategy was deemed appropriate as these systems did not align with the more recent conceptualizations of what an EHR includes. In general, there has been an increase in the literature on this topic since 2004. Interestingly, many of the issues and topics identified in the *Results* section do not appear to be constrained to a certain period. We would expect to see advancements in EHR infrastructure being reflected in the themes and issues raised in the included studies. This lack of visible change may be because of the low reporting of EHR functions and technical features, limiting the opportunity to see major trends in how EHRs have advanced over time in the mental health context. Table 2 outlines the key topics and publication dates of the included studies.

#### EHR System Used

We noted the name of the EHR and whether it was custom built or off the shelf. We also assessed whether the EHR functions or technical details had been reported. We did not expect all the studies to report this information, such as studies drawing on the secondary analysis of data. We first identified studies that expected to report the details of the EHR in their methods sections, such as the evaluations of specific EHRs (28/40, 70%) [78,79,81-84,87-89,91,92,95,99-111,114,115,117]. Of these 28 studies, 16 (57%) either named the EHR or provided details as to whether it was custom built or off the shelf [78,79,83,84,89,95,99-102,105-108,110,117]. One of the studies pointed to other publications in which the details of the EHR were reported [103]. Of the studies that reported details of the EHRs used, we tried to establish whether they were off-the-shelf commercial EHRs or custom-built EHRs. Some papers did not provide these details, and we searched for further information on the web to categorize those EHRs. Of the 16 studies that reported on the EHR, 3 (19%) were custom built [89,106,117], 12 (75%) were off the shelf [78,79,83,84,95,99-102, 107,108,110], and 1 (6%) was unclear [105]. The common off-the-shelf models were RiO [84,99,100,107,108] and EPIC [95,110]. Some studies outlined that commercial off-the-shelf EHRs have been adapted for the mental health context, such as through the addition of mental health–specific modules [102,110]. However, most studies did not clearly state whether and how off-the-shelf EHRs had been customized for the local context.

Of the 28 studies that we expected to report EHR details in their methods sections, only 7 (25%) discussed the functions of the EHR [79,81,84,89,103,105,110]. Sometimes, functions could be assumed from the results sections. No studies reported on the technical aspects of an EHR. Owing to the limited reporting of EHR types and functions, a comparison across studies was not feasible. The only theme that arose from these studies was that in the United Kingdom, many National Health System services used the same EHR (RiO), whereas, in the United States, there was more variety.

Several studies involved the collection of data on the type of EHRs that services were using, such as cross-sectional surveys of health services. We expected these studies to report details of the EHR in their results sections (8/40, 20%) [80,85,96,97,112,113,116]. Of these 8 studies, 3 (38%) reported on the names or types of EHR used by the included health services [97,112,116], and 2 (25%) reported some details of the functions [80,112], which were mainly related to those who could access the EHR. The included studies reported a variety of off-the-shelf and custom EHRs. For example, in the survey by Cellucci et al [116], they found that most psychology clinics used a commercial system, whereas a small number used custom-designed systems. Another example was the survey by Wu et al [97], which found 17 different off-the-shelf EHRs used by services in the US National Drug Abuse Treatment Clinical Trials Network. Multimedia Appendix 1 lists the EHRs reported in each study.

#### Theory

The included studies rarely referred to any underlying theory being used. Of the 40 studies, 2 (5%) of studies that reported using theories from the field of information behavior [104,105], 3 (8%) studies reported using sociotechnical theories [99,107,108], and 2 (5%) studies used the Technology Acceptance Model [110,116]. Approximately 3 (8%) of the 40 other studies also discussed the use of different theories [78,79,115].

#### Quality of the Studies

Scoping reviews do not incorporate an evaluation of the quality of the included studies, although some authors may consider it appropriate to do so [68]. This scoping review included a diversity of studies that no single evaluation method could appropriately address. A significant quality issue that we identified was the lack of detail regarding the EHRs, such as their functionality. The quality criteria for health informatics papers by Talmon et al [118] recommend studies that include information about the system in use.

## Results

### Overview

In the following sections, we report the findings of the textual narrative and thematic synthesis of the 40 included studies. The analysis led to the development of 6 main themes and several subthemes. The quotes that support the themes are provided in Multimedia Appendix 2 [79,81,86,88,98-100,104,105,107, 110,111].

### Supports Better Management of Most Information

This theme relates to how EHRs were found to support certain information practices, such as documentation and information accessibility. However, although EHRs show improvements over paper records, there are still issues with the completeness of documentation, especially of mental health information.

#### Documentation of Information

Several studies reported an improvement in the completeness of documentation in EHRs compared with paper records [78,87-89,102,113-115]. Electronic documentation also addressed issues of legibility that were common in paper records [88,89,99,108]. Improved documentation may partly be because of EHRs promoting accountability in documentation practice and prompting clinicians for certain information [87,105,114]. Approximately 2 (5%) of the 40 studies suggested a greater coupling of policy and practice guidelines within EHRs compared with paper records as the guidelines could be embedded in the EHR, such as through templates [79,105]. These templates provided less discretion regarding how information collection policies were followed. Although EHRs improved documentation compared with paper records, they still showed poor documentation of certain information [87,95]. Tsai and Bond [87] found that past psychotropic medications, prior hospitalizations, and clinical outcomes were regularly missing in EHRs. Bell et al [117], in scanning an EHR to identify drug- and alcohol-related issues, discovered that relevant information was more likely to be found in free-text progress notes, although structured forms were available. An interesting issue raised by participants in the study of EHR use in an integrated care trial by Cifuentes et al [112] was that new types of health care professionals could bring new types of data that the EHR was not designed to collect.

Some studies have found that EHRs create conditions that might negatively affect the documentation. Ser et al [100] found, in interviews with staff across 2 mental health hospitals, that long delays can occur between information collection and documentation in the EHR. Meredith [78] found, from a survey of community mental health teams, that both an EHR and paper record were used side by side, leading to some information not being documented in the EHR.

The benefits of improving documentation came with an increased time burden for clinicians [100,101,103,111,113]. This time burden was related to issues such as simple documentation tasks requiring multiple steps in the EHR [103]. Matthews [101] found that templates may speed up documentation but create challenges if clinicians need to navigate multiple screens and menus. Increased time spent documenting information in EHRs may lead to time savings when reviewing clinical notes in the future [81,110]. For example, Bhe et al [81] reported that 97% (28/29) of primary care physicians who had received access to psychiatric notes in the EMR reported increased efficiency in encounters with psychiatric service users.

#### Missing Mental Health Data

Several studies found that mental health information was regularly missing from EHRs, documented in the wrong place, or underdocumented in specific contexts [93,95-98,106]. For example, Gleeson et al [91] found that relying on diagnostic codes in an EHR would have missed 92.4% (110/119) of the mental health diagnoses. However, the information needed to make a diagnosis was available in other parts of the record. The same issue was found in the US Veterans Affairs EHR, where 40.9% (45/110) of people with a posttraumatic stress disorder diagnosis did not have it recorded [96]. Similarly, Madden et al [95] found that many psychiatric services for people with diagnoses of depression or bipolar disorder were missing from the EHR data when compared with health insurance claims. Gibson et al [104], in exploring how clinicians search for information in an EHR, found that when information is not present, clinicians may assume the opposite. For example, if the information on noncompliance with treatment is not present, clinicians may assume that the service user is compliant.

There are many reasons why mental health information may be missing in EHRs. Zhou et al [106] found that psychosocial information may be communicated verbally between clinical team members and not recorded in an EHR immediately, if at all. This practice may be because of psychosocial information being viewed as too subjective to be initially recorded in the EHR [106]. Wu et al [97] found that substance use disorders were not thoroughly captured in EHRs, partly because of the continued use of paper records for that specific part of the health service. Furthermore, in non–mental health services, mental health–related information collection may occur informally and may not be officially recorded in the EHR [106]. Madden et al [95] found that missing mental health data could result from service users seeking mental health care outside their regular health service. Missing information may also be because of the stigma, as discussed further in the following sections.

#### Access and Availability of Information

The use of EHRs appeared to improve legibility, timely access, and the availability of information [84,87,89,99,108,109,115]. These improvements allowed information to be found more easily when responding to concerns or issues [99,115]. The availability of information also benefited administrative staff, such as health information managers, who could easily look up mental health information [102]. Improved access to information was also viewed as contributing toward safer and higher quality care [99,102,116]. Boyer et al [103] reported that 74.8% (86/115) of health care professionals interviewed in a psychiatric hospital reported improved access to service user information with an EHR. However, not all information is available on EHRs [112]. Clinicians may have to go through a complex process of identifying what information they need and where they can access that information [104]. Clinicians may also struggle with navigating the EHR because of the amount of information it contains, which is an issue when EHRs do not include search functions [104,106].

Finally, information may be collected for several purposes. The availability of information for one purpose, such as providing care, may not necessarily mean availability for another purpose, such as reporting [100]. Larrison et al [94] found that for community mental health agencies, "capturing data to improve reporting capabilities" was a key motivation for implementing an EHR.

### Creates New Structures That Shape Information Collection

This theme reflects the finding that the adoption of the EHRs introduced new structures that shape information collection. These structures standardize and formalize information collection and raise several issues, especially in the mental health context, where unstructured narrative information is used extensively.

#### Standardized Information

The issue of data standardization arose in several articles, where data fields in the EHR were not suitable for mental health information. Structured fields cannot easily capture the *gray* narrative information common in mental health contexts, and trying to fit data into structured fields can have implications for care [101]. Two common issues were restrictive templates that took away from the narrative format of mental health notes [101,113] and essential templates or data fields missing from the EHR [99,106,112]. Common information collection forms used for mental health care, such as care plans and mental health screening tools, were missing in several EHRs [101,109,113]. When forms were missing, individual clinicians had to decide how to record the information [106]. In some cases, clinicians created standalone tools, such as spreadsheets to collect data. However, this further fractured information in EHRs, unless work was undertaken to integrate the information [112]. EHR formats not being suitable for the mental health context also led to data being captured in other parts of the record, such as free-text boxes or laboratory value areas, which can affect future uses of the data [100,101,109,117]. In addition, the extensive use of free text can make EHRs challenging to navigate [109].

Some of the reasons why standardization did not suit the mental health context included the level of personalization needed in the mental health contexts [111] and that some mental health information is subjective and could be perceived in different ways by different health services [106]. The use of diagnostic codes in an EHR may also create extra work when service users do not clearly fit any one diagnostic code [99]. Specific models of care may also require greater flexibility and personalization of the information collected [111]. An example of this is found in a study on person-centered care planning by Stanhope and Matthews [85,111], who found that standard forms in the EHR were barriers to person-centered care.

Standardization is not necessarily a negative process, and Takian et al [99] found that the standardization of letters sent to people’s general practitioners was viewed as beneficial. Clinicians have also recognized the benefits of data management tools to improve the searchability, visibility, and accessibility of information [103,108].

Standardization was also raised as a broader systems issue, where EHRs could not be tailored to specific organizations or settings. This issue was raised in a few studies that adopted commercial EHRs [101,110,111]. In a series of studies from the UK National Health Service (NHS), where uniform EHRs were being adopted, services wanted to tailor the standard solution to their unique needs and the changing priorities of their communities [99,100,107,108].

#### Informal Versus Formal Documentation

The 8% (2/40) of studies that explored the process that clinicians go through to document information found an element of informality in how mental health information was collected before a specific judgment was made and recorded in the EHR [105,106]. Hardstone et al [105] described how mental health clinicians used informal information practices to develop ideas before they were formalized in the health record. Paper records appeared to enable this informal documentation. In contrast, this provisionality enabled by paper records is limited by EHRs, where the information entered is viewed as a finalized account. Compared with a paper record, recording in an EHR had a greater sense of finality and permanence, which did not align with the informal discussion and sharing of assessments in integrated care settings [105]. Hardstone et al [105] outlined how EHRs may tightly embed rules around who can access records and when, which limits the flexibility to work on notes collaboratively. Zhou et al [106] found that EHRs did not have the functionality to capture provisional information.

### Supports Information Sharing and Communication

This theme captures how EHRs supported the components of integrated care, including information sharing and communication among professionals.

#### Communication Among Service Providers

The specific functions of EHRs may support information sharing and communication among service providers. The functions of EHRs that improve communication include the ability to assign tasks or notes to other clinicians [104], the use of messaging systems [92,101], and shared care plans [112]. These functions that allow clinicians to share information about service users can support the tailoring of care, reduction of unnecessary assessment, and reduction in the number of times service users have to retell the theory story [101]. However, not all EHRs had these functions [112]. There is some evidence that EHRs can improve service users’ experience of integrated care. Hu et al [90] found that EHR adoption was significantly associated with improved service user experience for "care transition" and "discharge information" in psychiatric hospitals. Jetelina et al [110] also found a significant improvement in service users’ perceptions of integrated care after the implementation of a mental health–specific EHR. However, EHRs that support integrated care may have to be situated in a model of care [85].

#### Interoperability Between EHRs and Services

Interoperability was raised as an issue across several contexts in the included studies. Several papers acknowledged that integrated EHRs are not always linked with all relevant mental health services [99,100,107,115]. An issue raised in implementing a national EHR in the UK NHS [100,107] was drawing boundaries regarding what services and clinicians can access the EHR. Ser et al [100] outlined how some local community services’ information systems were not integrated into the EHR, although these services played a significant role in providing mental health care. Robertson et al [107] also acknowledged that individuals may receive care from many services that are not always contained within a specific geographic region, which an EHR was designed to include. Furthermore, some EHRs lacked interoperability within and among health services [112]. Workarounds for the lack of interoperability identified by Cifuentes et al [112] included printing information from one EHR and scanning it into another EHR or duplicating documentation, which created delays and extra work.

### Disrupts Information Management Workflows That Affect the Therapeutic Relationship

This theme explores how EHRs disrupt information practice workflows and raise concerns regarding therapeutic relationships.

#### Workflow Disruption

Nonalignment of EHRs with workflows was raised in several studies [84,100,103,108,116]. For example, 34.6% (9/26) of psychology training clinics represented in the study by Cellucci et al [116] raised "the difficulty of getting EMR to do what they wanted" as a barrier to implementation. Boyer et al [103] found that 73% (84/115) of interviewed health care professionals in a psychiatric hospital raised the issue of workflow in connection with reduced efficiency, specifically, the challenge of balancing service user care needs and using the EMR. Workflow misalignments led to less time for direct care, which was viewed as affecting the therapeutic relationship [87,100,103].

Sheikth et al [108], Takian et al [99], and Edwards et al [84], in examples of the RiO EHR from the UK NHS, outlined how mental health presentations were complex and varied and required long and detailed assessments. Participants raised that it would not be feasible to try and get people in a crisis setup near a computer so that they could input notes simultaneously [108]. This situation may lead to information having to be inputted later, which could have a broader impact on the operations of the hospital [108]. Participants in the study by Ser et al [100] outlined the challenge of balancing EHR use and supporting people in a crisis, which is common in the mental health context. In clinical therapy, Matthews [101] found that some specific psychological therapies that are more structured may be appropriate for EHR documentation, such as cognitive behavioral therapy.

Matthews [101] and Ser et al [100] found that the EHR interface and design were more medically orientated and designed for contexts in which service users could be treated and discharged and did not need ongoing care. They also found that EHRs missed key mental health functions such as treatment planning and mental health screening. Workarounds were developed to overcome these EHR issues; however, they could be time consuming and require extra work [101]. In comparison, participants in the study by Jung et al [102] who used an EHR specifically designed for mental health contexts commented that they appreciated the EHR being designed for their workflow, including multidisciplinary documentation functions. Administrative staff, including health information managers, valued the ability to make changes to the templates in the EHR where necessary [102]. Similar findings were apparent in the research by Jetelina et al [110], where a mental health–specific add-on to an EPIC brand of the EHR system was evaluated. The tool improved screening and had good acceptability by clinicians.

#### The Therapeutic Relationship

The findings regarding the impact of EHRs on therapeutic relationships were mixed. Stewart et al [82] found no significant impact on the therapeutic relationship in a survey of people accessing outpatient psychiatric services where EHRs were used. In interviewing health care professionals at a psychiatric hospital, Boyer et al [103] found that 47% (54/115) were concerned about the triangulation of the therapeutic relationship with the inclusion of an EMR. Interestingly, Matthews [83] found that clinicians rated EHRs as more disruptive to care than service users did. This difference could be explained by the finding that clinicians used a number of strategies to integrate EHR into a session to minimize disruption for service users [83]. Conversely, EHRs have been seen as strengthening the therapeutic relationship by opening the documentation process to service users for discussion and better tailoring care to service users’ needs [101].

#### User Design, Computer Literacy, and the Learning Curve

Several studies have reported that EHRs’ complex user interface designs contributed to workflow disruption [99,101,102,111,113,115]. This complexity was related to navigating multiple screens and menus and working with complex templates. Matthews [101] found that clinicians had to navigate various parts of the EHR (screens, menus, and tabs) to record information and that templates did not always follow a structured order that was relevant to the session’s progress. Some of these issues may also be specific to the type of clinician. Jung et al [102] found that nurses who had the broadest range of access within the EHR experienced confusion because of the number of modules and the amount of information available to them. Issues with user interface design led to increased time burden for clinicians when documenting information in the EHR [100,101]. Alerts in the EHR were raised as issues in 8% (2/40) of the included studies [102,115]. Some studies reported frequent system crashes or technical glitches such as server issues, which severely affected EHR use and care provision [100,101,111,113]. Participants in the study by Takian et al [99] reported issues logging in and out of systems, especially as legacy systems were running alongside the EHR.

Low computer literacy was raised as a reason why clinicians may find the user interface of the EHR complex [100-102]. Clinicians may also have variable computer skills, specific skills such as typing, and general skills in using technology [88,100-102,107]. For some clinicians, the learning curve can be quite significant [110]. Sheikh et al [108] also found that EHRs may be designed for one type of clinician rather than for many health care professionals and administrative staff using the EHR. Several studies raised the importance of high-quality training to address usability issues [99,102,116].

### Challenges Clinician’s Management of Sensitive Information

This theme relates to how EHRs raise issues regarding the management of sensitive information and how reducing access to certain parts of the EHR was a common approach to managing issues of sensitive information.

#### Sensitive Information

Several studies acknowledged that information collected in the mental health context could be particularly sensitive, such as information on traumatic personal events [80,89,93,97,103,113]. EHRs may lead to sensitive information collected by clinicians being more widely available to other clinicians, thus challenging the confidentiality between service users and clinicians [88,100,103]. Several studies explored how specific conditions, including posttraumatic stress disorder [96], substance use [97], mental health diagnoses among people living with HIV [98], and sexual trauma [93], were documented in EHRs. These topics were generally contextualized as sensitive, which affected their documentation. In studies that explored clinicians’ documentation practices, an approach clinicians took to sensitive information was generalizing it or *watering it down* [89,100]. Another approach was excluding this information from the EHR or keeping a *shadow record* or paper record for mental health information [89,96]. A finding from the study by Zhou et al [106] points to the subjectivity in clinicians’ decisions regarding when to document mental health information.

In some cases, concerns about sensitive information were related to a lack of clarity regarding the legal requirements regarding privacy and confidentiality [113] and the need for further training on these topics [116]. Psychiatric health care professionals in the study by Boyer et al [103] raised the issue of balancing the need to record sensitive information for the provision of care with the risk that it may be used to create a *profile* of service users for other purposes.

#### Mutual Access to Psychiatric Information

A common indirect way that sensitive information was raised as an issue was by sectioning mental health records in the EHR [80,86,89,97,113]. By sectioning the record, nonpsychiatric clinicians could not access mental health notes or could only access them with a password or if they were willing to *break the glass* and have their access recorded. For example, Bhe et al [81] reported that psychiatrists were given the option of creating two separate notes in the EHR, one accessible by other psychiatric clinicians and one for nonpsychiatric clinicians.

There is evidence that mutual access to psychiatric information supported the provision of mental health care. Bhe et al [81] found that primary care clinicians valued access to psychiatric information as it enabled them to provide care relevant to someone’s psychiatric needs, such as by considering the side effects of medication. Mutual access to mental health records may also support care coordination between mental health care and primary health care providers [86,113]. Colaiaco et al [86] found, in practices with a mutual EHR, that 46% (19/41) of reviewed service users’ primary care records showed some contact between primary health care and mental health care clinicians compared with only 11% (11/100) in practices with no mutual EHR. Furthermore, 100% (24/24) of the reviewed records in services with a mutual EHR had medication information updated across mental health and primary care providers’ records compared with 57% (31/54) in nonmutual EHR services.

This study does not seek to consider the clinical implications of EHRs. However, we would be remiss not to mention a finding from the study by Kozubal et al [80] that there was a significant relationship between increased accessibility (nonpsychiatric clinicians’ ability to access psychiatric records) and reduced readmission rates.

### Raises Legal Concerns for Clinicians Regarding Their Information Responsibilities

The final theme regarding legal issues, particularly those related to privacy and mental health laws, appeared in far fewer studies than we had anticipated. There was little congruence among the references to legal concerns, with a variety of different concerns raised across the included studies. Reitz et al [113] found that the use of EHRs raised concerns about compliance with relevant information privacy laws. Ser et al [100] found that clinicians expressed concerns about whether EHRs aligned with their requirements under relevant mental health legislations. In the study by Jung et al [102], administrative staff, such as health information managers, outlined how the EHR supported compliance with relevant regulations by reducing the reporting burden. Clinical staff also reported wanting alerts relevant to their legal requirements when people were being treated under the relevant mental health laws [102]. Participants in the survey by Cellucci et al [116], representing psychology training clinics, identified the need for training on ethical issues, confidentiality, and security standards. Participants in the study by Matthews [101] outlined how state regulations and standards shaped the design and use of EHRs.

## Discussion

### Principal Findings

This scoping review aimed to explore how EHRs in the mental health context affected the information practices of health care professionals and how these changes affected other aspects of care. Issues relevant to the mental health context, such as the management of sensitive information, data standardization, and therapeutic relationships, were also explored. We found that EHRs can improve some information practices but need to be designed appropriately for specific workflows and information types in the mental health context. Beyond the design of EHRs, the redesign of health service workflows and clinician training may be needed to ensure that EHRs can be used effectively in the mental health context. Information collected in the mental health context is considered more sensitive than other types of clinical information. Greater guidance may be needed regarding how sensitive information is managed in EHRs to ensure that it is documented and used appropriately. In the following sections, we consider how the findings of this review link back to the broader literature on EHRs.

The documentation of clinical information is a critical information practice that informs current and future care [119-121]. The findings of this review point to improvements in the relative quantity of the information documented when using an EHR compared with paper records. However, information was still missing from EHRs, which may affect future care. Furthermore, a common issue for clinicians was the inflexibility of the fields in EHRs and the time required to input data. This issue may be partly because of the greater coupling of policy and process with tools for documentation, such as templates. Mamykina et al [121] has raised this focus on templates and structures in EHRs as an outcome of viewing clinical documentation as a *composition* activity. However, through a time-and-motion study, they found that clinical documentation was a synthesis activity involving clinicians accessing several informal and formal information sources that they synthesized into clinical documents. This reflects the finding from this review that informal documentation is a necessary precursor to formal documentation and contributes to the synthesis of the final documentation. Mamykina et al [121] argued that tools for composition, such as templates, differ from tools for synthesis, which should promote access to various information sources, such as informal notes that previously could be written and edited within the paper document. This finding may explain why certain information is missed in the structured documentation in EHRs, although it was available in other free-text sections.

The focus on the standardization and the formalization of documentation exposed a critical tension between current approaches to health informatics and contemporary mental health care. An objective of EHRs is the standardization of health information to allow for health information exchange and data analytics [122,123]. In comparison, mental health care involves the documentation of a large amount of narrative information, much of which resists standardization [16,51]. An increasing focus on recovery models of mental health care that prioritize service user–defined measures and outcomes may create further tensions with standardized data collection [124]. Concerns have also been raised about EHRs impeding clinicians’ ability to understand a service user’s entire story [125]. These issues were discussed in 1998 regarding the need for an informatics framework specific to mental health [126]. Future research and EHR design need to establish which standardized information is relevant for the mental health context and how best to present narrative information to capture service users’ stories.

The issue of standardization found in this review is not unique to mental health in that paper records, in general, provide more opportunities than EHRs for recording narrative information [127]. The many benefits promised by EHRs in terms of decision support, streamlined reporting, and supporting research are premised on the need for structured data entry [24]. However, narrative information is highly valued by clinicians. This may reflect why clinicians used narrative information, even when structured fields were available. Our findings and research in other contexts indicate that clinicians prefer documentation methods that align with their workflows and allow them to record more details about clinical encounters [24,128-130]. Narrative information provides opportunities for clinicians to convey information such as uncertainty, unique aspects of cases, and nuances in the service user’s appearance, which is not supported by structured documentation [24]. A potential solution to the tension between unstructured and structured documentation is the application of software to unstructured clinical notes that can extract relevant data into structured fields. For example, natural language processing could be applied to free-text narratives to fill structured EHR fields [24,127].

The management of sensitive information was raised as a key concern in the adoption of EHRs. The definitions of and overlap between sensitive information and mental health information are unclear. The National Committee on Vital and Health Statistics [131] outlined the complexity of defining mental health information in that it can be collected in a variety of clinical settings and may be scattered throughout a person’s health record. Data about physical health, collected in mental health settings, may also be considered mental health information. However, there appears to be a subsection of mental health information classified as *sensitive* for several reasons, such as the stigma related to certain diagnoses. There is also a relationship between standardization and sensitive information, with some studies in this review finding that sensitive information may be captured in free-text notes but not in standardized fields. Perhaps free text provides more nuances in documenting this type of information. For example, Cairns et al [132], in a study of social workers using a shared record, found that they had concerns about reporting subjective information that other people could wrongly interpret. One of the potential issues with incorrect or vague documentation in the mental health context is that it could feed into incorrect risk assessments [133]. Risk assessments in mental health can have significant implications for people’s health outcomes and their human rights if a risk assessment leads them to be involuntarily treated [133].

Shared decision-making has become a key approach for promoting autonomy in health care decision-making, especially in the mental health context [134]. This can be seen in the practices of clinicians inviting service users to be involved in deciding what information to document in their health record, which is known as collaborative documentation [91]. Inviting service users to participate in decisions about what information goes into their EHR and how to document sensitive information could help address concerns that clinicians might have about privacy or stigma. Pisciotta et al [135] found that clinicians and service users in mental health settings avoided discussing notes because they worried about each other’s responses. Pisciotta et al [135] also found that service users want clinicians to be open to discussing what is written about them and have opportunities to collaborate in documenting information. Collaborative documentation may also address concerns about the therapeutic alliance if workflows are redesigned to accommodate EHRs and service users. Maniss and Pruit [136] outlined how collaborative documentation involves clinicians documenting service user information alongside service users and creating opportunities for their input and approval. However, as was found in the included studies, the current EHR design is not aligned with the complexity of some mental health contexts where service users may arrive in a crisis. Thus, the adoption of collaborative documentation may need to happen alongside other service changes to ensure that EHRs can be more easily integrated into service users’ care.

The findings related to the relationship between information practices and therapeutic relationships require more research, especially from the service user perspective. It has been suggested that most information practices are invisible to service users [137] unless there are active efforts to make them visible. However, these practices and how they are shaped using EHRs will affect service users’ experience of care through impacts on the therapeutic relationship or the time available for direct care. Much research has focused on service users’ perspectives concerning the privacy and confidentiality of EHRs [12]; however, the actual impact on the experience of care has received limited attention. There is a growing body of evidence exploring the role of computers in clinical encounters, which may capture some of these experiences [138,139]. The impact of computers on the interaction between clinicians and service users can be shaped by factors such as the clinician’s skill in using the computer and the way clinicians embed computers in their practice [140]. Findings from the study by Pearce et al [141] showed that computers had become part of a *triadic relationship* with clinicians and service users, which is not necessarily a negative outcome. Future work should explore how EHRs as sociotechnical systems affect the care provided and service users’ experience of these impacts.

### Comparison With Prior Work

#### Overview

There are several reviews related to different elements of EHRs, which generally support the findings of this review. In a systematic review of the impact of EHRs on documentation time, Baumann et al [23] found that EHRs were associated with increased documentation time for hospital staff. The interaction between service users and clinicians was also raised as potentially threatened by the use of EHRs [142]. Workflow issues were also identified by Gephart et al [143] in a systematic review of nurses’ experiences of EHR. They found that EHRs created unexpected changes in the accepted workflows. Strudwick and Eyasu [144], in a review of the literature on EHRs used by nurses in mental health settings, also identified the unique nature of the mental health context. They found that nurses were conscious of the privacy and confidentiality risks posed by the ease of access enabled by EHRs. A recent scoping review on EMR implementations in mental health settings by Zurynski et al [30], which also included studies in children and adolescents and several review studies not specific to the mental health context, also found issues with documentation, workflows, and usability.

The issue of usability that was raised in this study has been confirmed by previous reviews exploring navigation in EHRs [143,145,146]. Roman et al [145] found that navigation between EHR screens was a regularly identified usability issue that could be addressed through shortcuts, dashboards, and integration of information into single screens. Training has also been found to enable the acceptance and use of EHRs [146,147]. McGinn et al [142], in a systematic review of barriers to and facilitators of EHR implementation, also found that usability could be both a barrier to and a facilitator of EHR use.

An increasing number of studies have identified new secondary uses for the data collected in EHRs [148]. These secondary uses include applications in psychiatric phenotyping [149] and methods for predicting suicidal behavior [150]. The potential impact of this secondary data use makes it increasingly urgent to address the issues raised in this study. Secondary data use in the mental health context requires further ethical consideration, especially as new data sources are being introduced into the health care system, such as data from wearables [151,152].

#### Relevance of Findings for EHR Designers

One of the key issues identified in this review was that EHRs were not appropriately designed for the mental health context. Thus, we will target our recommendations for those who design and develop EHRs.

Designers must ensure that they understand clinicians’ information practices in the mental health context. There are examples of EHR usability frameworks such as the TURF (task, user, representation, and function) framework [153], which can guide EHR design. A key point raised by the TURF framework is the need to understand the complexity of a task independent of how it is implemented in a specific setting. Our review found that many EHRs were not designed to address the complexity of the mental health context. This issue could be because of designers and developers not understanding the essential elements of certain tasks and how these should be represented in the design of the EHR. Our review also found that many EHRs are missing data fields relevant to mental health and provide limited ways of managing narrative data. Thus, improving the customizability of EHR workflows may be useful. Alternatively, several preset workflows could be provided for different types of service users or clinical contexts. The study by Jetelina et al [110] provides an example in which an add-on for an EPIC EHR was developed containing specific features for the mental health context. Designers should also consider the computer literacy of their end users and what relevant training and support may be needed.

The time burden experienced by clinicians when documenting information in EHRs raises questions about the systems’ user experience design. This time burden is not just an issue in the mental health context, with O’Brien et al [154] describing the broader issue as *death by data entry.* This is a critical issue for clinicians and is associated with increased odds of burnout [155,156]. This could be addressed in several ways, including through research, policy initiatives, and design methods [157]. Our findings suggest that further research is needed for workflows in the mental health context and how EHR functions can support rather than disrupt these workflows. Addressing this issue should also lead to greater end user involvement in designing, developing, and implementing EHRs in the mental health context [158]. Improving clinician training may also support the use of EHRs [99,100].

#### Relevance of Findings for Clinicians

A key issue found in this review was the poor documentation of mental health information in EHRs. Missing information is detrimental to both the care of service users and clinicians’ work. It appears that there is a perception among clinicians that mental health information, being particularly sensitive, should be documented differently from other information. We would advise clinicians to consider approaches such as collaborative documentation in which service users are involved in discussions about what to document. If there is doubt about how to word certain sensitive information, clinicians should ask the service user and consider the implications for future clinical encounters and the service user’s experience if certain information is missing or misinterpreted.

#### Relevance of Findings for Health Service Managers and Health Policy Makers

From this initial evidence on EHRs in the mental health context, it would be advisable for health service managers to scope their options when adopting an EHR. Services should start by identifying their information and workflow needs before choosing an EHR. Some EHRs designed specifically for the mental health context are more appropriate than generic EHRs. Otherwise, specific add-ons that meet the workflow and information needs of the mental health context may be considered. Furthermore, well-executed training is necessary to ensure that clinicians have appropriate computer skills to manage the complex user interfaces that some EHRs present.

We would advise policy makers to support the adoption of EHRs only when they are designed for local contexts. In Australia, the Victorian Royal Commission into Mental Health Services has recently recommended that information systems should be used to improve care in the mental health context [159]. We would advise that further research is needed to identify the mechanisms by which EHRs will lead to the assumed outcomes and any barriers or structural issues to achieving these assumed outcomes.

#### Relevance of Findings for Service Users

It was concerning that there was minimal involvement of service users in the included studies. The issues identified in this review will have implications for service users. These impacts may be related to disrupted workflows or sensitive information being recorded incorrectly. Many service user groups are taking great interest in the digitization of the health system, and we would encourage them to continue this involvement, especially with a focus on EHR development.

#### Relevance of Findings for Future Research

Future researchers should report on the types and functions of the EHRs they are studying. This would enable greater comparison between contexts. Adopting a standard approach to describing EHRs such as the Health Care Information and Management Systems Society’s [160] EMR Adoption Model may support comparison across studies. Furthermore, more details about the setting of the research should be provided. Health information technology is a global business, and companies providing EHRs to the United States also provide them to other countries. Providing more details about the setting of implementation and the type of EHR would support evidence synthesis that other jurisdictions can also rely on.

Future research should also include service user perspectives on EHRs and information practices. Researchers should consider adopting co-design or participatory methods to involve service users in research about EHRs. It would also be advisable to involve peer workers within health services in the design of EHRs as they may see how these EHRs have been used in practice. Researchers should also involve more health information managers and other administrative staff. These stakeholders play a critical role in supporting the correct management of information in health care settings.

The field of research on EHRs in the mental health context is still at a low stage of maturity, and this, in part, reflects the maturity of EHR use in the mental health context. Future research should include high-quality evaluations of EHRs in the mental health context for both implementation and sustained use. This research will pave the way for systematic reviews that can provide insights into how EHRs affect processes and clinical outcomes in the mental health context. We would also recommend further studies on the usability of EHRs or that usability analysis be included in other study designs.

Notably, we could not conduct a temporal analysis of the included papers. Recent decades have seen considerable advancements in the fields of health informatics and digital health [161]. It would make sense that these advances should be reflected in the included papers. We might expect to see improvements in interoperability because of the increasing adoption of solutions such as the Fast Health care Interoperability Standard [162]. We may also expect to see improvements in the documentation of standard information using clinical terminologies such as the Systematized Nomenclature of Medicine [163]. However, these advances were not discussed in the included papers. We can speculate why this was the case. It might be that these innovations have not penetrated the mental health context or affected the issues we have identified. However, what is needed in future research is a greater focus on the technical aspects of digital health research. Future studies should aim to report the technical aspects of EHRs in practice to enable greater visibility of how EHR innovations penetrate real-world applications.

Finally, a further piece of research that should be considered is how digital health or information system theory can accommodate the findings of this review and others related to the use of EHRs. A few of the included studies drew upon the theory in their work; however, more work could be conducted to extend this work. We have reflected that many of our findings could be considered using an *Activity Theory* lens, and we would welcome discussions and collaborations to further this thinking.

### Limitations

This scoping review is limited, in part, because of the nature of the field. The combination of no standard EHR definition and poor reporting of the systems used in the included studies has made it difficult to assess how specific themes related to specific types of EHRs. This review examines information practices, which is one of the many potential research topics that could be addressed in this space. Other studies should examine clinical outcomes. We expected to find more studies reporting on legal and ethical concerns, and in hindsight, a more tailored search may have been needed. There was limited information on the technical aspects (such as interoperability standards) of the EHRs used in the included studies. This limited information affected our ability to comment on whether the technical elements of the EHR contributed to our findings. The United States’ focus of the included studies also limits the applicability of the findings to other jurisdictions, especially those related to health system structure and culture.

### Conclusions

EHRs in the mental health contexts have been slow to materialize. This review found that EHRs in the mental health context affect clinicians’ information practices, which have implications for how care is provided. The core of mental health services is the therapeutic relationship, which requires a unique workflow that is currently not supported by many EHRs. In addition, because of the narrative nature of mental health care, the standardized data underpinning many EHRs may not align with the information needs and practices of the mental health context. Finally, although health information is recognized as personal information, mental health information is seen as especially sensitive for several reasons. This understanding of mental health information may lead to underreporting, generalizing, or watering down certain details when documenting in EHRs. EHRs need the capacity to support information sharing in a nuanced way to manage sensitivity and stigma in the mental health context. Future research should involve service users to explore how the impact of EHRs on information practices also affects their experience of care.

## Appendix

Multimedia Appendix 1 Included studies.

Multimedia Appendix 2 Quotes to support themes.

## Abbreviations

EHR: electronic health record
EMR: electronic medical record
NHS: National Health Service
PRISMA-ScR: Preferred Reporting Item for Systematic Reviews and Meta-Analyses extension for Scoping Reviews
