# Supplementary material for: Impact of Electronic Health Records on Information Practices in Mental Health Contexts: Scoping Review
Source: J Med Internet Res. 2022 May 4;24(5):e30405. doi: 10.2196/30405 (PMC9118021; doi:10.2196/30405)
Supplement: Multimedia Appendix 1 [file jmir_v24i5e30405_app1.docx]

**Multimedia Appendix 1: Summary of included Studies.**

| **Reference** | **Country** | **Study type** | **Study Design**  *Categorized by authors* | **Study Design and Method**  *Summarised by authors* | **Context/Setting** *Summarised by authors* | **EHRs Discussed** [Where we expected EHR details to be reported]  *Summarised by authors* | **Focus / Aim**  *Summarised by authors* |
| --- | --- | --- | --- | --- | --- | --- | --- |
| Bell et al.,  2013 [116] | UK | Mixed | Chart review | Scanning of acute admission records and comparing them with patient interviews. | Acute admission wards - Maudsley Hospital | Electronic patient journey system [Method] | Assessing the usefulness of an electronic patient record and whether it has improved the identiﬁcation and response to alcohol- and drug-related problems. |
| Bhe et al.,  2015 [80] | US | Quantitative | Survey | Survey of primary care physicians. | UC Davis Medical Center Campus | Not described [Method] | Assessing how primary care physicians utilize psychiatric documentation in an EMR. |
| Boyer et al.,  2010 [102] | France | Qualitative | Interviews | Interviews with hospital staff and health care professionals. | Teaching psychiatric hospital | Not Described / Described in another paper. [Method] | Exploring health care professionals’ opinions of the opportunities and barriers of the implementation of an EMR in a teaching psychiatric hospital. |
| Brown et al.,  2020 [97] | US | Quantitative | Secondary analysis/cross-sectional | Data extracted from electronic medical records. | Center for AIDS Research | NA | Reporting rates of psychiatric diagnoses among persons living with HIV in an EMR. |
| Cellucci et al., 2015 [115] | US | Mixed | Survey | Online survey of clinical directors of psychology training programs. | Doctoral psychology training programs which were members of the Association of Psychology Training Clinics. | Titanium Scheduler, CarePaths, Point and Click, & Custom designed systems. [Results] | Assess current EMR use in psychology training clinics. |
| Cifuentes et al., 2015 [111] | US | Mixed | Ethnographic | Observational comparative study of primary health clinical and community mental health clinics who were implementing integrated care. | Primary care practices and community mental health centers. | e-MDs V7.2, GE Centricity V9.5, eClinical Works V10.0, Allscripts TouchWorks V11.4, Health Connect (EPIC) V Summer 09, NextGen V5.8, Siemens EDM V24, Siemens LCR V2.2, Document management systems and clinical database, Unicare Profiler V15, AdvancedMD, Netsmart TIER, & Qualifacts Systems, Inc., Carelogic V5, Intergy. [Results] | Describing the experience of using an EHR across primary and behavioral health clinics implementing integrated care. |
| Cofiel et al.,  2013 [108] | Brazil | Case-study | Descriptive case-study | Case-Report | Tertiary psychiatric institution | Not described [Method] | Case-study report of an EHR implementation in a tertiary mental health institution |
| Colaiaco et al., 2018 [85] | US | Quantitative | Chart review | Review of medical records | Six geographically diverse Medicare Advantage plans. | NA | Comparing the continuity of information between mental health and primary care clinicians with and without an EHR. |
| Edwards,  2011 [83] | UK | Quantitative | Survey | Survey of mental health nurses regarding their attitudes towards electronic records | Acute and forensic wards | RiO [Methods] | Exploring mental health nurses’ views about the contribution of electronic records to care planning with patients. |
| Gibson et al., 2016 [103] | US | Qualitative | Interviews | Observation and think aloud interviews of clinicians interacting with an EHR and follow-up interview. | Outpatient mental health clinic of George E. Whalen Salt Lake City VA Medical Center. | Not described [Method] | Describing how clinicians search for information related to adherence in an EHR. |
| Gleeson et al.,  2016 [90] | Ireland | Quantitative | Secondary analysis/cross-sectional | Reviewing the EMR of a random sample of patients across seven general practices. | General practices | Not described [Method] | Using an EHR to identify prevalence and treatments of mental illness in primary care settings. |
| Hardstone et al., 2004 [104] | UK | Qualitative | Ethnographic | Ethnographic study of community mental health teams. | Adult and Care of the Elderly Community Mental Health Teams | Integrated Care Planning Pathway Database [Method] | Describing the formal and informal character of information sharing practice in community mental health teams. |
| Hu et al.,  2020 [89] | US | Quantitative | Secondary analysis/cross-sectional | Cross-sectional study secondary analysis from two public data sets on hospitals in the United States. | American Hospital Association Annual Survey Database containing information for 6,251 hospitals | NA | Exploring the relationship between the adoption of certified EHRS in psychiatric care settings and patient experiences. |
| Huerta,  2015 [91] | US | Quantitative | Survey | Survey of health professionals at a mental health clinic. | Mental health clinic | Not described [Method] | Exploring the satisfaction of health professionals towards an EHR. |
| Jetelina et al., 2018 [109] | US | Mixed | Surveys & interviews | Proof-of-concept studying involving the implementation of a set of behavioral health tools in an EHR across six community health centres. | Community Health Centers | EPIC [Method] | Exploring whether a set of custom designed behavioral health tools for an EHR changes the process of care, patient experiences, and clinical outcomes, as well as its acceptability to clinicians. |
| Jones et al.,  2019 [92] | US | Quantitative | Secondary analysis/cross-sectional | Extraction of data from clinical notes using natural language processing | National, random sample of Veterans who served in recent conflicts and who received care in Veteran Health Affairs medical facilities | NA | Describing variations in how sexual trauma is documented in EHRs |
| Jung et al., 2021[101] | US | Qualitative | Interviews | Interviews with health care professionals and administrative staff across four behavioral health hospitals. | Behavioral hospitals | BestCare 2.0B [Method] | Exploring the experiences of mental health professionals using an EHR – with a focus on barriers and facilitators. |
| Kozubal et al.,  2013 [79] | US | Quantitative | Survey | Survey of psychiatrists at top US hospitals and comparison with clinical outcomes data. | Psychiatrists at 18 of the top US hospitals | Not described [Results] | Exploring the use of EMR by psychiatrists in US hospitals, and their relationship to patient care outcomes. |
| Larrison et al., 2018 [93] | US | Quantitative | Survey | Cross-secondary analysis of a national survey of community mental health agencies. | Community mental health agencies | NA | Examining the implementation of EHRs among community mental health agencies. |
| Lipford et al.,  2017 [54] | US | Mixed | Survey | Survey of clinicians in an inpatient psychiatric hospital who used the EHR. | Inpatient psychiatric hospital | Not described [Method] | Exploring facilitators and barriers to EHR use in an inpatient psychiatric hospital. |
| Madden et al., 2016 [94] | US | Quantitative | Secondary analysis/cross-sectional | Comparison of insurance claims data with data extracted from EHRs. | Members of Harvard Pilgrim Health Care assigned for primary care to Harvard Vanguard Medical Associates, a multispecialty medical practice | EPIC [Method] | Comparison of behavioral health data in EHR with more complete insurance data. |
| Martin et al.,  2018 [113] | Canada | Mixed | Chart review | Review of nursing documentation of PRN medications compared with verbal reports. | Public psychiatric hospitals | Not described [Method] | Describing and comparing documentation of pro re nata medication for anxiety between a hospital using paper charts and a hospital using an EHR. |
| Matthews,  2017 [100] | US | Qualitative | Interviews | Cross-sectional, qualitative study involving interviews with behavioral health clinicians. | Community health center network providing integrated health and behavioral health services | “Popular commercial EHR system” [Method] | Explore experiences of behavioral health clinicians with experience of using an EHR in face-to-face treatment encounters. |
| Matthews,  2020 [82] | US | Quantitative | Survey | Cross-sectional sample of 35 clinical sessions. Video recordings and post-visit survey of both service users and clinicians. | Large, urban federally qualified health center offering integrated health and mental health services | “Common EHR system” [Method] | Exploring service users and clinicians perceived impact of EHRs on in-session communication. |
| Matthews & Stanhope,  2020 [84] | US | Quantitative | Survey & chart review | Chart reviews of service plans across three time points. | Community mental health clinics | Not described [Results] | Examining the influence of EHR availability on the fidelity of person-centered care planning in mental health contexts. |
| Meredith,  2009 [77] | UK | Quantitative | Survey | Validated questionnaire sent to clinical staff working in community mental health teams. | Community mental health teams | In4Tek’s PAIS system [Method] | Evaluating the deployment of electronic patient records in community mental health teams. |
| Reitz et al.,  2012 [112] | US | Mixed | Survey | Mixed-method – survey. | Collaborative care offices where medical and mental health clinicians provide collaborative care. | Not described [Results] | Reporting on the effects of EHRs in primary care settings that are integrated with behavioral health services. |
| Riahi et al.,  2017 [78] | Canada | Quantitative | Quality improvement | Case-study | Tertiary-level mental health facility | Meditech 6.0 [Method] | Documenting the implementation and customization of an EMR in a tertiary mental health facility. |
| Robertson et al., 2010 [106] | UK | Qualitative | Ethnographic | Mixed-methods longitudinal multisite case-studies, | National Health Service acute hospital and mental health trusts | RIO [Method] | Describing and evaluating the implementation and adoption of electronic health records in secondary care. |
| Salomon et al., 2009 [88] | US | Quantitative | Survey | Survey of clinicians who had recently switched to an EHR. | Vanderbilt Medical Center Department of Psychiatry | Custom EHR system [Method] | Understanding psychiatric clinicians’ views of recently implemented EHR. |
| Ser et al.,  2014 [99] | UK | Qualitative | Interviews | Interviews with clinical, information technology, managerial and other staff across two mental health hospitals who were early adopter of a national EHR. | Mental health National Health Service hospitals | Rio [Method] | Investigating perceptions and reported practices of mental health hospital staff using national hospital EHR. |
| Sheikh et al.,  2011 [107] | UK | Qualitative | Ethnographic | Longitudinal qualitative evaluation using several case-studies of hospitals who were early adopters of a national EHR. | NHS acute hospitals and specialist care settings | Cerner Millennium, RiO & Lorenzo RegionalCare [Method] | Evaluating implementation and adoption of EHRs in secondary care hospitals. |
| Stanhope & Matthews,  2019 [110] | US | Mixed | Interviews & quality improvement | Focus groups, interviews, and analysis of implementation reports. | Community mental health clinics | Not described [Method] | Exploring how EHRs and EHR stage of development influenced implementation of person-centered care planning. |
| Stewart et al.,  2010 [81] | US | Quantitative | Surveys | Pre-test and post-test survey of service users accessing people who were outpatients of a psychiatric clinic. | University of New Mexico Psychiatric Center Continuing Care Clinics. | Rio [Method] | Examining the association between EHR use and changes to patient-psychiatric relationship. |
| Takian et al., 2012 [98] | UK | Qualitative | Ethnographic | Longitudinal, real-time, case-study-based evaluation of the implementation of an EHR. | Mental health hospital | Not described [Method] | Describing the arrival, implementation process, experiences, and consequences of an EHR implementation. |
| Tsai & Bond, 2007 [86] | US | Quantitative | Chart review | Comparison of paper medical record to EHR records through a chart review process. | Community mental health centers | Not described [Method] | Examining whether EMRs improve medication documentation and retrieval compared to paper records |
| Wilk et al.,  2016 [95] | US | Quantitative | Survey | Web-based survey of army mental health providers. | Army mental health providers | Not described [Results] | Identifying extent to which posttraumatic stress disordered diagnoses are record by army mental health providers in their EHR, reasons for not recording and implications. |
| Wu et al.,  2020 [96] | US | Quantitative | Survey | Survey of members (clinicians) of the National Drug Abuse Treatment Clinical Trial Networks who have knowledge of their facilities EHR. | A range of healthcare facilities affiliated with the National Drug Abuse Treatment Clinical Trials Network | EPIC, Avatar/Myavatar, Cerner, Allscripts, Meditech, Centricity Practice Solutions, Centricity, Netsmart CMHC/MIS, Professional EHR, Awards, Canopy, Hyperspace-Prod, Menon, Nextgen, Point and Click, Success EHS, Welligent. [Results] | Exploring the use of EHRs and paper records to capture clinical care tasks related to substance use disorders. |
| Xiao & Acosta, 2016 [87] | US | Quantitative | Quality improvement | Quality improvement reviews of medical record post-implementation | Large public  psychiatric outpatient | Not described [Method] | Describing the implementation of EMRs in a psychiatric outpatient clinic to improve clinical documentation. |
| Zhou et al.,  2010 [105] | US | Qualitative | Ethnographic | Field-based study at a large teaching hospital. | General medicine services in the department of internal medicine at a teaching hospital. | In-house developed EHR [Method] | Examining clinicians use and documentation of service user information for care. |
