# Supplementary material for: Impact of Electronic Health Records on Information Practices in Mental Health Contexts: Scoping Review
Source: J Med Internet Res. 2022 May 4;24(5):e30405. doi: 10.2196/30405 (PMC9118021; doi:10.2196/30405)
Supplement: Multimedia Appendix 2 [file jmir_v24i5e30405_app2.docx]

**Multimedia Appendix 2: Illustrative quotes that support themes.**

| **Themes and Sub-Themes** | **Illustrative Quotes** |
| --- | --- |
| **Supports Better Management of Most Information.** | |
| - Documentation | *“Two-way analysis of variance analyses found highly signiﬁcant main effects of documentation on completeness and retrieval time, showing that documentation in electronic records were signiﬁcantly more complete and faster to retrieve than paper records across the three sites…”[86]* |
|  | *“…we can observe that a closer coupling is afforded between policy and electronic documents….Thus organizational ‘rules’ can be more directly and tightly embedded, leaving fewer opportunities for profession discretion to be exercised.”[104]* |
| - Missing Mental Health Information | *“For this case, even though the psychosocial issue emerged as a main concern, it still did not seem legitimate enough to be documented in the record. As one resident stated, “You never know for sure.” [105]* |
| - Access and Availability | *“Enhanced availability of data and data management tools were perceived as benefits by managers and some clinical users when information was legible, available in “real time,” more easily searchable and retrievable (such as for management purposes), and accessible “any time” and “anywhere” by multiple concurrent users” [107]* |
| **Creates New Structures That Shape Information Collection.** | |
| - Standardized Information | *“Record keeping is also very different from the approach used in the acute sectors as the notes tend in mental health settings to be more narrative in nature.” [98]* |
|  | *“Both clinics experienced difficulty personalizing information due to drop down menus giving them uniform options rather than the ability to individualize goals and objectives. One provider described the problem, acknowledging the benefits of uniformity but also showing how this can conflict with the spirit of person-centered care” [110]* |
| - Informal Versus Formal Documentation | *“Many other psychosocial issues critical to understanding a patient’s needs and motives are also shared only verbally without being documented. This leaves the next care team in an information vacuum and requires the repetition of time-consuming investigations in complicated patient conditions.” [105]* |
|  | *“On one occasion, a team member was observed to erase text written in pencil from the ‘Outcomes’ section of the form…She stated that her reason for this was that she had written down her ideas about how the patient should be managed – but that she anticipated that following discussion with the rest of the team…some of her “ideas” would be taken up, some would not, and others might emerge during the course of the meeting.” [104]* |
| **Supporting Information Sharing and Communication.** | |
| - Communication Between Service Providers | *“EHRs typically did not have templates that supported shared care plans for both primary care and behavioral health needs. However, EHRs that had tasking functions were helpful in enabling some types of communication and coordination between team members.” [111]* |
| - Interoperability Between EHRs and Services | *“…concerns were raised by these interviewees about the lack of integration between the hospital EHR system and other IT systems used locally by, for example, NHS psychologists and local authority social workers who were also involved in the care of their patients.”[99]* |
| **Disrupts Information Workflows that impact the Therapeutic Relationship.** | |
| - Workflow Disruption | *“Many interviewees were of the view that the EHR system did not integrate well with their existing work practices and required more time of them to use.” [99]* |
|  | *“…being designed on a simplistic, linear interpretation of the workflows in mental health settings. They thus criticized the software as not reflecting the contextual differences across care settings.”[98]* |
| - The Therapeutic Relationship | *“…we found no change in satisfaction scores among adult, psychiatric patients when an EHR was used during outpatient encounters instead of paper charting.” [81]* |
| - User Design, Computer Literacy & the Learning Curve | *“While seemingly insigniﬁcant, providers related that these series of clicks actually accounted for a meaningful amount of time.” [100]* |
|  | *“Consequently, most interviewees preferred to learn through using the software in practice rather than in traditional classroom environments.” [98]* |
| **Challenge Clinician’s Management of Sensitive Information.** | |
| - Sensitive Information | *“Many indicated the use of “measured” …or generic wording... For example, the review of side effects may be thoroughly recorded, while speciﬁc aspects of childhood incest traumata might be mentioned only as “inappropriate contact”” [88]* |
|  | *“This case showed how the medical team, when they felt it appropriate, would document the psychosocial information for a patient. Clearly, this case was unusual. It highlights, nonetheless, the emphasis on the doctors’ sense of ‘appropriateness’ in determining when to document.”[105]* |
| - Mutual Access | *“While all sites had electronic records in at least one*  *category, not all hospitals gave non-psychiatric physicians unrestricted access to at least one type of psychiatric note.” [79]* |
| **Raises Legal Concerns for Clinicians** | *“Importantly, the EHR system at that time was perceived not to*  *meet clinical needs with regard to regulations specifically relating to mental health, including national mental health legislation.”[99]* |
